# Retinal Periphery Is Insensitive to Sudden Transient Motion

**DOI:** 10.1177/2041669520937029

**Published:** 2020-06-28

**Authors:** Stuart Anstis

**Affiliations:** Department of Psychology, University of California San Diego

**Keywords:** motion, noise, peripheral vision, illusion

## Abstract

Peripherally viewed targets moved around against a background of random dynamic noise. Slow movements were visible, fast movements were not. Thus, a target that repetitively drifted to the right and snapped back appeared to drift endlessly to the right with no visible snapbacks.

We report a novel illusion of peripherally viewed motion. Movie 1 shows two red squares, each of diameter 3°, in motion both with and without a randomly twinkling background. The upper square jumps back and forth at 2 Hz in apparent motion between two positions 0.6° apart (dwell time in each position is 250 ms). The lower square repetitively drifts to the right for 500 ms through a distance of 0.6° (drift speed = 1.2°/s) and snaps back again to the left. So the position over time of the upper square describes a square wave and of the lower square describes a repetitive sawtooth. The random dynamic noise in the background attenuates the motion by reducing the signal to noise ratio.

In foveal vision, observers see the motion accurately. But in peripheral vision, especially when the random noise is present, an illusion appears; the upper square seems to flicker in place with almost no perceptible side-to-side motion, and the lower square appears to drift continuously (and paradoxically) to the right without getting anywhere, and without visibly snapping back. Thus, the slow drift in the lower square is still visible in peripheral vision but the sudden transient jumps in both squares, when attenuated by the random noise, are not.

**Movie 1.** View in peripheral vision. The upper square jumps back and forth but appear to flicker in place. The lower square slides right and snaps back repetitively, but appears to keep sliding continuously to the right. So in peripheral vision you see the slow but not the fast motions.

In Movie 2, the upper red circle moves along an X-shaped path. It drifts up to the right through a distance of 0.6°, jumps down, drifts up to the left, and jumps down again to its starting point: and repeats this cycle endlessly. Each drift lasts for 300 ms, so the cycling rate is 1.7 Hz and the drift speed is 2°/s. In foveal vision, it is clear that it jiggles around within a small area. But in peripheral vision, the downward vertical jumps are barely seen, so the circle appears to zigzag upwards endlessly. The lower circle follows an inverted X, so that it appears to zigzag endlessly downwards. Thus, when viewed peripherally, the two circles appear to move continuously further and further apart, although the overall physical distance between them never increases.

**Movie 2.** View in peripheral vision. The circles each jiggle along X-shaped paths, but they seem to be constantly receding from each other. So again, in peripheral vision you see the slow but not the fast motions.

The rapid jumps are not perceived in peripheral vision, and no countermanding position signals restore veridicality. Without the attenuating random noise, the fast and slow phases of the motion in these movies are both detectible and the illusion of continuous drifts is only very slight, as shown in Movie 1. Since dynamic random noise produces a constant barrage of motion signals in all directions, its masking effect in these movies may be a form of motion crowding.

This loss of peripheral sensitivity to rapid motion does not indicate a loss in *spatial* resolution, since the slow motions can still be seen in the periphery. Instead, the limited *temporal* resolution of the periphery may be curtailing its response to speedy motion that is weakened by noise, so the fast transient jumps are too rapid to be registered.

These findings are at variance, if not in direct conflict, with some well-established results. For instance, McKee and Nakayama (1984) measured the peripheral detection of shear between adjacent visual stimuli and concluded that while velocity discrimination was spatially M-scaled, temporal sensitivity was more or less constant across the retina – whereas our results suggest that the periphery is more sluggish than the fovea. Foster et al. (1989) presented two closely spaced points in rapid sequence and obtained an illusion of a single dot moving through a greater distance than the points’ actual separation. They dubbed this the fine-grain movement illusion, and it occurred at separations of the two points that were smaller than the static two-point thresholds. Thus, they observed a greater extent of peripheral motion than the stimuli seemed to warrant, while we found a smaller extent. We cannot explain these discrepancies, beyond noting that our stimuli and conditions are very different from those in the works cited.

Others have reported anomalous motion perception in the periphery. In the furrow illusion (Allard & Faubert, 2016; Anstis, 2012), a moving target is correctly seen in foveal vision, but appears when viewed peripherally to move parallel to a background of tilted stripes. Tse and Hsieh (2006) reported an infinite regress illusion, in which a tangentially moving global window appears to move continually away from fixation, even though it remains a fixed distance from fixation. The window is filled with bars moving away from the fovea, which indicates motion away from fixation, and are incorrectly attributed by the visual system to the motion trajectory of the whole window (Anstis, 1989).
